# Georgia Medical Association

**Published:** 1883-09-20

**Authors:** 


### *GEORGIA MEDICAL ASSOCIATION.*

A writer in the Atlanta Medical Register complains of the $3.00 assessment upon members, and thinks that a smaller fee—the Transactions to be printed in a cheaper form—would result in bringing out more members by encouraging the village and country Doctors to join, and would be productive of much good.

If the Society would publish the Transactions in the SOUTHERN MEDICAL RECORD, it would cost much less money and would reach ten to one of its present readers. It could be done in a single issue as a Supplementary Department of the Journal, and in one or two months after the adjournment

